# Evaluation of Different Attractive Traps for Capturing Sand Flies (Diptera: Psychodidae) in an Endemic Area of Leishmaniasis, Southeast of Iran

**DOI:** 10.18502/jad.v14i2.3739

**Published:** 2020-06-30

**Authors:** Saideh Yousefi, Ali Reza Zahraei-Ramazani, Yavar Rassi, Hassan Vatandoost, Mohammad Reza Yaghoobi-Ershadi, Mohammad Reza Aflatoonian, Amir Ahmad Akhavan, Abbas Aghaei-Afshar, Masoumeh Amin, Azim Paksa

**Affiliations:** 1 Department of Medical Entomology and Vector Control, School of Public Health, Tehran University of Medical Sciences, Tehran, Iran; 2 Sirjan School of Medical Sciences, Sirjan, Iran; 3 Department of Chemical Pollutants and Pesticide, Institute for Environmental Research, Tehran University of Medical Sciences, Tehran, Iran; 4 Research Center for Tropical and Infectious Diseases, Kerman University of Medical Sciences, Kerman, Iran; 5 Leishmaniasis Research Center, Kerman University of Medical Sciences, Kerman, Iran; 6 Department of Parasitology, Faculty of Medicine, Tabriz University of Medical Science, Tabriz, Iran

**Keywords:** Control, Leishmaniasis, Plant component, Chemicals, Iran

## Abstract

**Background:**

The attraction of phlebotomine sand flies to plant and animal hosts is due to the produced chemical compounds, affecting the olfactory receptors of the insects. Therefore, novel and effective methods, such as Attractive Toxic Sugar Baits (ATSB) and Attractive Toxic Baits (ATB), are based on the effective materials that attract sand flies toward the host. The present study was designed to identify the attractive materials in plants and animals for using in ATSB and ATB.

**Methods:**

This cross-sectional study was carried out in July 2018 on endemic areas of leishmaniasis in Iran. Different baits, including mango, nectarine, grape, banana, melon and watermelon, defibrinated blood of cattle, sheep, goat and chicken, urine of cattle, sheep, goat and ultimately, simple and complex chemicals, such as CO_2_, 1-octanol, lactic acid and human sweat were placed inside the traps, and the rate of the sand flies attraction to these materials was studied. Furthermore, data were analyzed using the Kruskal-Wallis test and Mann Whitney U test.

**Results:**

There was a significant difference in the sand flies attraction between the traps containing watermelon, urine of cattle, and sheep, and chemicals such as CO_2_ and human sweat and the control trap (p< 0.05).

**Conclusion:**

This study showed that watermelon and CO_2_ are the potential candidates for using in ATSB and ATB, respectively.

## Introduction

Some species of phlebotominae sand flies (Diptera: Psychodidae) transmit a group of metaxenic diseases, such as leishmaniasis, sand fly fever, summer meningitis, vesicular stomatitis, chandipura virus encephalitis, and carrion disease (1).

In terms of mortality and morbidity, leishmaniasis is the most important diseases transmitted by some species of sand flies (*Phlebotomus* and *Lutzomyia* genera) and they are also among the 9 most important infectious diseases in the world (2). Leishmaniasis mainly occurs in tropic and sub-tropic regions of Africa, America, Asia, and Europe continents (3, 4). Almost half of the countries in the Eastern Mediterranean Region (EMRO) of the World Health Organization (WHO) are endemic foci of cutaneous and visceral leishmaniasis. Iran, along with Afghanistan, Iraq, Saudi Arabia, Syria, Yemen, and Pakistan are the major foci of Anthroponotic Cutaneous Leishmaniasis (ACL), and *Leishmania tropica* is the causative agent in these areas (5).

Kerman, Tehran, Khorasan, Fars, Yazd, and Esfahan provinces are the main active foci of ACL in Iran. Researches have proven that in these foci, *Phlebotomus sergenti* is the main vector, *L. tropica* is the causative agent, human is the main and dog is the secondary reservoir hosts (6, 7).

Bam County is the most infectious focus of ACL in Kerman Province (8). After the earthquake in 2003, which resulted in the inhabitants’ homelessness and increased breeding places for sand flies, epidemics of this disease were reported in the city and several rural areas which previously were free of leishmaniasis (9).

Currently, the main prevention activities for controlling the disease in Bam County, are case diagnosis and treatment with glucantim, health education, and occasionally, indoor residual spraying (10, 11). There is no effective vaccine against leishmaniasis. The usual drug (glucantime) is expensive and has several side effects on heart, kidney, and liver (12). Indoor residual spraying not only increases the vector resistance to insecticide, but also is expensive and has side effects on the environment and non-target organisms (13). Up to now, controlling the vector populations is the most effective way to control metaxenic diseases, including leishmaniasis (14).

Attractive Toxic Sugar Bait (ATSB) is an effective and environmentally friendly method for controlling phlebotomine sand fly populations. In this method, attractive plant substances, such as, fruit juice combined with an oral insecticide, are used and this compound attracts and kills insects. ATSB can be sprayed on non-flowering vegetation or it can be used as bait station, and this controlling method has low side effects on non-target organisms (honey bees, beetles, etc.) and environment (13). In the studies carried out in Iran, Morocco and Israel (Jordan Valley), sand fly populations were reduced using this method (13, 15, 16).

Attractive Toxic Bait (ATB) uses simple or complex chemicals produced by the host body (human and animal) such as CO_2_ (in animal breath), lactic acid and octanol (in host sweat), urine, sweat, and blood to attract the insects. Such compounds have been introduced as effective materials in attracting Diptera (17–22).

Considering the WHO emphasis on identifying affordable and effective methods for use in Integrated Vector Management (IVM) programs (12), this study examines the rate of phlebotomine sand flies attraction to traps using different baits containing fruits such as mango, nectarine, grape, banana, melon and watermelon, the defibrinated blood of cattle, sheep, goat and chicken, the urine of cattle, sheep, and goat, as reservoir hosts, and simple and complex chemicals such as CO_2_, 1-octanol, lactic acid, and human sweat.

## Materials and Methods

### Study area

**Fig. 1.**
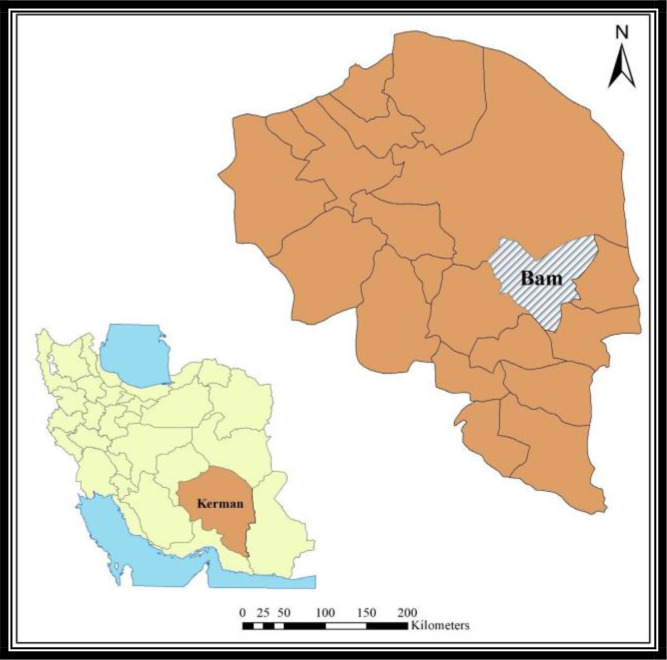
The study area in Bam County, Kerman Province, Iran, 2018

This study was conducted in July 2018, in Bam County (29° 06′.52″ N latitude and 58° 22′.67″ E longitude, 1050m above sea level) of the Kerman Province, Iran (Fig. 1). The topography of the studied area is lowland plain. This area has hot and dry summers and temperate winters, the mean annual temperature, precipitation and relative humidity is 25 °C, 68mm, and 20%, respectively. Palm (*Phoenix dactylifera*) is the most common tree in the region (23).

### Study on the rate of the sand flies attraction to different baited traps

#### Traps containing different fruits

Mango (*Mangifera indica*), nectarine (*Prunus persica*), grape (*Vitis vinifera*), banana (*Musa sapientum*), melon (*Cucumis melo*) and watermelon (*Citrullus lanatus*) were used as attractive baits for trapping sand flies based on the method used by Mong’are et al. (22), with changes in the appearance of the trap.

The trap consisted of two main components: A 5-liter plastic bottle and a Castrol oil-impregnated paper at a size of 29×20cm, which was glued to the inner wall of the bottle by the castor oil.

**Fig. 2.**
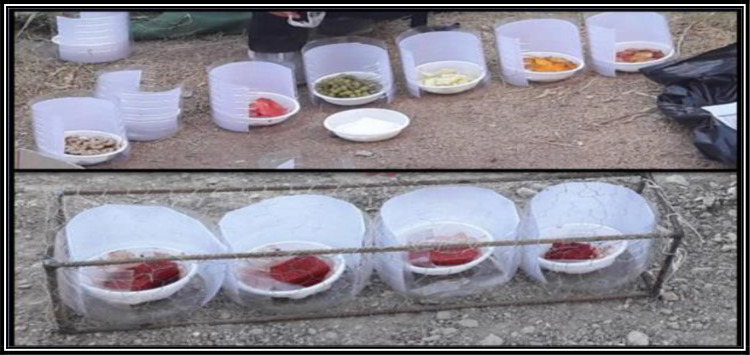
Baiting traps for attracting sand flies

The upper and one of the lateral parts of each bottle were cut to provide the sand flies with access to the fruits (Fig. 2). Also, a container was placed on the bottom of the bottle for holding the fruits. The fruits were bought from local stores, 200g of which was daily placed inside the trap. The negative controls included a trap containing a water impregnated sponge, a trap containing sucrose solution 10%-impregnated sponge, and an empty trap.

These traps were placed in various biotopes for 10 days in July 2018, from 6:00PM to 6:00AM, and to minimize the location error in the sampling, the traps were rotated every day through different locations. The distance between the traps was at least 2m.

#### Traps containing the defibrinated blood of animals

Blood samples of cattle (*Bos taurus*), sheep (*Ovis aries*), goat (*Capra aegagrus*), and chicken (*Gallus gallus*) were freshly collected from slaughterhouses and they were defibrinated using glass balls. The bottles containing defibrinated blood were kept in the refrigerator. A total of 200ml of the animals’ blood was transferred into the container and a sponge with an appropriate size was inserted into the container to absorb all the blood. Then, the sponge was placed inside the trap. To prevent the access of wild animals to the blood sponges, the traps were placed inside metal nets designed for this purpose. The traps were placed in domestic, peridomestic, agricultural, and sylvatic biotopes for 10 days in July 2018. Moreover, 2 negative controls were used in this stage, including a trap containing water impregnated sponge and an empty trap.

#### Traps containing the urine of different animal taxa

The urine of cattle, sheep, and goat, prepared from local livestock was used in this study. For this purpose, 200ml of animal’s urine was placed inside the container and a sponge was inserted to absorb all the urine; then the sponge was placed inside the traps. The research was performed in 4 different environments. Two controls were used in this stage, including traps containing water-impregnated sponge and empty ones

#### Traps containing simple and complex chemicals

In each biotope, 200g of dry ice (CO_2_) was used inside the traps. The traps were rotated every day through different locations. Furthermore, 16 bright nylon socks, that were worn by children (girls and boys) and adults (men and women) for at least 24 hours, were used in each trap (approximate weight: 200g), in order to investigate the rate of phlebotomine attraction to human sweat.

The sweaty socks were placed in 4 different biotopes for 10 days, and new pairs of socks were replaced by the old ones in each sampling.

In order to determine the rate of phlebotomine sand flies attraction to 1-octanol and lactic acid, a sponge impregnated with 200ml of pure 1-octanol and lactic acid was placed inside the traps. Also, 2 controls, including water-impregnated sponge trap and an empty trap, were considered for the investigations.

#### Identification of the captured sand flies

The sand flies collected from each trap were stored in ethanol (96%). The identification was based on the morphological characteristics of the male (genitalia and cibarium) and female (spermathecae and pharynges) using the morphological key by Seyedi-Rashti et al. (24).

### Statistical analysis

The normality of the quantitative variable (attraction rate) was investigated by the Kolmogorov-Smirnov test. Non-parametric Kruskal-Wallis test was used to study the difference between the median attractions among different traps. The Mann Whitney U test was also used to compare the attraction rate between each trap and control groups, and also to compare the control groups to each other. Statistical analysis was done by SPSS version 24.

## Results

### The composition of sand flies species

A total of 1651 sand flies specimens including, 14.3% female and 85.7% males, belonging to the 2 genera of *Phlebotomus* and *Sergentomyia*, were captured. The distribution of the captured sand flies was 66.87% in peri domestic, 18.83% in sylvatic, 13.45% in agricultural, and 0.85% in domestic sites. The relative abundance of each species was *Ph. sergenti* (73.53%), *Ph. papatasi* (5.87 %), *Sergentomyia baghdadis* (18.71%), *Se. sintoni* (1.39%) and *Se. dentata* (0.5%).

### The attraction of sand flies to traps containing various fruits

The results of the Kolmogorov-Smirnov test showed that the distribution of the desired variable (attraction rate) was not normal (p< 0.001). According to the Kruskal-Wallis test, the attraction rate was significant, especially in the female group (p< 0.001) (Table 1). The Mann Whitney U test indicated that the attraction rate was significant in the male group among all of the fruit and control traps. In the female group, there was a significant difference only between watermelon and the control trap (Table 1).

**Table 1.** Comparison of the sand flies attraction rate between different fruit and control traps in Bam County, 2018

**Sandfly sex**	**Fruit type**	**Median**	**Interquartile range**	**p-value**	**Comparison**	**p-value**
**Male**	*Mangifera indica*	8	11	0.018	*Mangifera indica* vs.^*^ water	0.009
	*Cucumis melo*	5.5	7.75		*Cucumis melo* vs. water	0.011
	*Musa sapientum*	5.5	9.25		*Musa sapientum* vs. water	0.023
	*Prunus persica*	9.5	11.25		*Prunus persica* vs. water	0.015
	*Vitis vinifera*	5	6.25		*Vitis vinifera* vs. water	0.023
	*Citrullus lanatus*	5.5	6		*Citrullus lanatus* vs. water	0.003
	Water control trap	2	4.25		Sugar solution vs. water	0.393
	Sugar solution control trap	3	4.25		Empty vs. water	0.739
	Empty control trap	2	4.5			
**Female**	*Mangifera indica*	1	4.25	< 0.001	*Mangifera indica* vs. water	0.218
	*Cucumis melo*	0.5	2.25		*Cucumis melo* vs. water	0.481
	*Musa sapientum*	0	0		*Musa sapientum* vs. water	0.068
	*Prunus persica*	0	0.25		*Prunus persica* vs. water	0.585
	*Vitis vinifera*	0.5	1.25		*Vitis vinifera* vs. water	0.529
	*Citrullus lanatus*	3	3.5		*Citrullus lanatus* vs. water	0.004
	Water control trap	0	1.25		Sugar solution vs. water	0.358
	Sugar solution control trap	0	0		Empty vs. water	0.486
	Empty control trap	0	0			

* vs. is the abbreviated form versus

### The attraction of sand flies to traps containing the defibrinated blood of different animals

The normality of the attraction rate was investigated by the Kolmogorov-Smirnov test. The results showed abnormality of the desired variable (p< 0.001). The difference between the median attraction rates were analyzed, using the Kruskal-Wallis test. The results showed that there was a significant difference in attraction rate only in the males group (p< 0.001) (Table 2).

**Table 2.** Comparison of the sand flies attraction rate between different blood and control traps in Bam County, 2018

**Sand fly sex**	**Type of blood**	**Median**	**Interquartile range**	**p-value**	**Comparison**	**p-value**
**Male**	Goat blood	4	5.25	< 0.001	Goat blood vs.^*^ water	0.004
	Sheep blood	4.5	4.5		Sheep blood vs. water	0.002
	Cow blood	5	7		Cow blood vs. water	0.001
	Chicken blood	2.5	3.25		Chicken blood vs. water	0.001
	Water control trap	1	1		Empty vs. water	0.77
	Empty control trap	0.5	1			
**Female**	Goat blood	0	0	0.313	Goat blood vs. water	-
	Sheep blood	0	1		Sheep blood vs. water	-
	Cow blood	0	0.25		Cow blood vs. water	-
	Chicken blood	0	2.5		Chicken blood vs. water	-
	Water control trap	0	1		Empty vs. water	-
	Empty control trap	0	0.25			

* vs. is the abbreviated form versus

Therefore, the Mann-Whitney U test was only used in this group to compare the attraction rate of each blood trap to the control, and also to compare the control groups with each other. The results showed that there was a significant difference in the attraction rate between the traps containing cow, chicken, sheep, and goat blood compared to the control trap (Table 2).

### The rate of sand flies attraction to traps containing the urine of different animals

In this study, the normality of the attraction rate variable was investigated by Kolmogorov-Smirnov test. The Kruskal-Wallis test was used to examine the difference in the median attraction rate between the different traps. There was a significant difference in attraction rate between male and female groups (p< 0.001).

Nonparametric Mann Whitney U test was used to compare the attraction rate of each sample (urine of different animals) to the control trap, and also to compare the control groups to each other. Results show that, in the male sand flies group, there was a significant difference in the attraction rate between the traps containing urine from goat, sheep, and cattle in comparison to the control trap (p< 0.05) (Table 3).

**Table 3.** Comparison of sand flies attraction rate between different urine and control traps in Bam County, 2018

**Sand fly sex**	**Type of urine**	**Median**	**Interquartile range**	**p-value**	**Comparison**	**p-value**
**Male**	Goat urine	5.5	16	< 0.001	Goat urine vs.^*^ water	0.001
	Sheep urine	6	14.5		Sheep urine vs. water	0.001
	Cow urine	7	8.25		Cow urine vs. water	0.002
	Water control trap	1	2		Empty vs. water	0.910
	Empty control trap	1	2			
**Female**	Goat urine	1	3	< 0.001	Goat urine vs. water	0.28
	Sheep urine	1	1.5		Sheep urine vs. water	0.045
	Cow urine	3	1.75		Cow urine vs. water	0.014
	Water control trap	0	1		Empty vs. water	0.436
	Empty control trap	0	0.25			

* vs. is the abbreviated form versus

In the female group; only the difference in the attraction rate between the traps containing the urine of sheep and cow was significant compared to the control trap (Table 3).

### The rate of sand flies attraction to traps containing various chemicals

The results of the Kolmogorov-Smirnov test showed that the attraction rate variable was abnormal.

Based on the Kruskal-Wallis nonpara-metric test, there was a significant difference in attraction rate between both male and female groups (p< 0.001) (Table 4). The results of the Mann-Whitney U test showed that only the differences in attraction rate between traps containing CO_2_ and human sweat were significant compared to the control trap (Table 4).

**Table 4.** Comparison of the sand flies attraction rate between different chemicals and control traps in Bam County, 2018

**Sand fly sex**	**Chemicals**	**Median**	**Interquartile range**	**p-value**	**Comparison**	**p-value**
**Male**	Human sweat	6	8.25	< 0.001	Human sweat vs.^*^ empty	0.007
	CO_2_	15.5	9		CO_2_ vs. empty	< 0.001
	Lactic acid	2	2.75		Lactic acid vs. empty	0.247
	1-Octanol	1	1.5		1-Octanol vs. empty	0.529
	Water control trap	1	2		Water vs. empty	0.631
	Empty control trap	1	1.5			
**Female**	Human sweat	1	2.25	< 0.001	Human sweat vs. empty	0.023
	CO_2_	2.5	5.25		CO_2_ vs. empty	< 0.001
	Lactic acid	0	1.25		Lactic acid vs. empty	0.28
	1-Octanol	0	0		1-Octanol vs. empty	0.99
	Water control trap	0	0		Water vs. empty	0.739
	Empty control trap	0	0			

## Discussion

Like the other blood-sucking vectors, sand flies need blood for egg development and, therefore they transmit some sand fly borne diseases to humans and animals (22). Identifying methods, for reducing the population of sand flies, decrease the burden of the disease, as well as economic and mental stress caused by these insects. Currently, in order to control leishmaniasis, chemical control, and environmental management are also used in addition to case finding and treatment (25). Also, using of some chemical control methods, such as indoor residual spraying (IRS), space spraying, insecticide-treated curtains, bed nets, and sleeping bags, not only increase vector resistance to insecticides but also have not been effective in some area, probably due to the limitation of contact with insecticides in these methods (26, 27, 15).

Moreover, successful environmental management methods require sufficient funds and facilities and complete knowledge on the ecological and biological characteristics of sand fly species in each region (28).

With the constraints and limitations of conventional sand fly control methods in mind, the World Health Organization (WHO) recommends the identification and evaluation of affordable and effective methods for using in Integrated Vector Management (IVM) strategies (12, 29). So far studies have reported the effectiveness of newly applied methods, known as ATSB and ATB in controlling sand flies and other vector families in different ecological regions around the world (17, 20, 30–32). Therefore it is essential to identify the most effective sources attracting vectors in each region in order to use them in ATSB and ATB.

Sugars are the only sources of energy for vital activities of male sand flies and one of the most important sources of energy for females. Therefore, sugar is an important and major factor in the life of these vectors, and one of the factors limiting the survival and fitness of these insects in dry areas is restricted sugar access (33).

Fruits, plant tissues, flower nectars, and aphid and coccid honey are the main sources of sugar for sand flies (33–35). Although, it is still not fully determined that which compounds produced by or derived from fruits attract the sand flies. However, the abundance of glucose, fructose, and sucrose has been proposed as one of the important sources of energy supply for sand flies various activities (36).

In order to study the effectiveness of the ATSB method in controlling mosquitoes (Culicidae), different fruit extracts combined with insecticides have been used and the success rate was reported as 36–100% (30, 37, 38, 39). Moreover, in studies conducted on the rate of sand flies attraction to different fruits, the highest rates have been reported for mango, banana, apple, and grape (22). According to a study conducted by Schlein (16), using nectarine extract, along with ingested insecticides, could reduce the population of sand flies, and thus this combination is useful in leismaniasis control programs. In studies carried out in Iran, it has been confirmed that using brown sugar as an attractant material, combined with boric acid and water for spraying on plants, and the application of fences treated with these materials against colonies of rodents, that are reservoirs of disease, are effective in controlling the population of *Ph. papatasi* as the main vector of ZCL in the Country (15).

Despite the importance of sugar in the life cycle of sand flies and the importance of fruits as one of the important sources of sugar supply, no studies have been yet conducted in Iran, on the rate of sand flies attraction to different fruits used in ATSB. Therefore in the present study fruits such as mango, banana, nectarine, grape, melon, and watermelon were used in traps designed for this purpose. Based on the results, watermelon (*Citrullus lanatus*) or its combinations are more attractive for male and female of sand flies, compared to other fruits. Thus, watermelon due to its ability to attract sand flies, especially males and females of *Ph. sergenti*, seems like a good candidate for using in ATSB.

In addition to the proper performance of ATSB in attracting flies, ATB containing materials from the host body has also been proven to be effective in deviating mosquitoes from the human host toward artificial baits containing these materials (40).

Female blood-sucking sand flies use CO_2_ to find the location of their host for sucking blood and therefore they are attracted to the traps containing this material. Aside from the female sand flies, male sand flies are also attracted to the CO_2_ emitted from the host body in order to find females for mating (41, 42). In Egypt, Italy, and Israel CO_2_ has been introduced as an effective attractive material for collecting sand flies (42–44). Hesam-Mohammadi et al. (45) showed that CO_2_ is not an attractive substance for capturing sand flies in Kashan district. In the current study, the highest rate of sand flies attraction, especially male and female *Ph. sergenti* as the main vector of ACL, has been reported in traps containing CO_2_, thus, it is clear that CO_2_ is one of the most effective substances for attracting sand flies in the region, and it can be effectively used in ATB to capture and control the population of sand flies or for entomological surveillance programs.

Studies have shown that the human body produces hundreds of fumigation molecules with simple and complex structures. Some of these materials can be used to attract medically-important insects (17, 18, 20). Various studies have shown that using socks worn by humans in traps can attract a great number of malaria vectors, and due to the persistence of the smell of sweat, closed areas containing worn clothes are good places for attracting *Anopheles* spp. mosquito and sand flies (19–21, 46).

Also, some compounds in human sweat, such as lactic acid, have been successfully used in malaria-vector capture programs due to their extensive attraction for mosquitos. In addition to the attraction of Diptera to lactic acid, octanol has also been proposed as an effective material in attracting *Nyssomyia neivai*, which is the vector of cutaneous leishmaniasis in the United State (19, 21).

A recent study showed that sand flies are attracted to human sweat; therefore, although the chemicals such as human sweat have been proven to be effective in attracting sand flies to humans and causing disease transmission, using this material in ATB can cause distraction of sand flies from humans to traps, and thus help reduce their population and diseases transmission.

Like many other blood-sucking insects, sand flies require proteins for their egg development, and animal and human blood are the most important supplies of protein (46). In studies conducted by Mong’are et al. (22), on the rate of the sand flies attraction to traps containing the blood of cattle, sheep, goat and chicken, the highest rates were reported for goat blood and the lowest was reported for chicken blood. This study demonstrated that, the male sand flies tend to be attracted to the blood of various animals, which seems to contain a material attracting male sand flies to the host for finding females. However, it seems that, the method used to attract sand flies to blood resources is not appropriate for female sand flies, or perhaps the preference of the female sand flies is to other hosts.

The animals’ urine containing, substances such as 4-methyl phenol and 3-n-propyl phenol, has been reported to be effective in capturing some of Diptera belonging to the Culicidae and Glossinidae families (47). In studies conducted by Keweka et al. (48), cattle urine was proposed to be effective in the attraction of mosquitoes belonging to the Culicidae family to breeding places containing this material for laying eggs. Few studies have examined the relationship between the host’s urine and its rate of sand fly attraction. According to studies in Kenya, the urine of cattle, goat and sheep is effective in attracting *Ph. martini*, *Se. africana*, *Se. antennata* and *Se. schwetzi* (22). The results of the recent study in Bam County showed that the urine of animals such as sheep and cattle attracts male and female sand flies, which is probably due to certain components emitted from the host’s urine as a guide for female sand flies to find a host location for laying eggs, and in the case of male sand flies, to find a proper site for mating. Therefore, it seems that these materials can be used in ATB to attract sand flies. As noted earlier, the largest number of sand flies was captured from peri domestic biotopes, which demonstrate that the animal kairomones are highly attractive for sand flies, and this biotope has been reported to be one of the most dangerous environments due to the sand flies accumulation and subsequently, diseases transmission to humans.

## Conclusion

The recent study showed that watermelon and CO_2_ were the potential candidates for using in ATSB and ATB, respectively. These two substances are more effective and affordable than other examined materials. The data obtained from this study can help to identify and introduce effective baits for using in Attractive Toxic Sugar Baits (ATSB) and Attractive Toxic Baits (ATB), in order to control the sand fly populations and reduce disease cases.
